# The minimal important difference of the pulmonary functional status and dyspnea questionnaire in patients with severe chronic obstructive pulmonary disease

**DOI:** 10.1186/1465-9921-14-58

**Published:** 2013-05-25

**Authors:** Eloisa MG Regueiro, Chris Burtin, Paul Baten, Daniel Langer, Hans Van Remoortel, Valéria A Pires Di Lorenzo, Dirceu Costa, Wim Janssens, Marc Decramer, Rik Gosselink, Thierry Troosters

**Affiliations:** 1 Postgraduate Programme in Physiotherapy, Universidade Federal de São Carlos - UFSCar, São Carlos, Brazil; 2 Special Unit of Respiratory Physiotherapy - UFSCar, São Carlos, Brazil; 3 Department of Rehabilitation Sciences, Katholieke Universiteit Leuven, Leuven, Belgium; 4 Respiratory Rehabilitation and Respiratory Division, University Hospital Gasthuisberg, Leuven, Belgium; 5 Postgraduate Programme in Physiotherapy, Universidade Nove de Julho - UNINOVE, São Paulo, Brazil

**Keywords:** Minimal important difference, PFSDQ – Pulmonary Functional Status and Dyspnea Questionnaire, COPD – Chronic Obstructive Pulmonary Disease, Pulmonary rehabilitation, Funtional status, Symptoms

## Abstract

**Background:**

The modified version of the Pulmonary Functional Status and Dyspnea Questionnaire (PFSDQ-M) is used in patients with COPD to obtain information about their functional status. It consists of 3 components (change in activities, dyspnea and fatigue) ranging from 0 to 100 and has been shown to be responsive following pulmonary rehabilitation (PR). The interpretation of changes in PFSDQ-M score after an intervention is difficult in the absence of the minimal important difference (MID) of the PFSDQ-M. This study aims at investigating the MID of the PFSDQ-M.

**Methods:**

We enrolled 301 patients with COPD (FEV_1_ 42 ± 15%pred) that completed the PFSDQ-M before and after a 3-month PR program (∆Chronic Respiratory Disease Questionnaire (CRDQ) +16 ± 12 points, ∆Six-minute walking distance (6MWD) +47 ± 89 m, both p < 0.001). An anchor-based approach consisted of calculating the correlation between the ∆PFSDQ-M and anchors with an established MID (∆CRDQ and ∆6MWD). Linear regression analyses were performed to predict the MID from these anchors. Secondly several distribution-based approaches (Cohen’s effect size, empirical rule effect size and standard error of measurement method) were used.

**Results:**

Anchor-based estimates for the different PFSDQ-M-components were between −3 and −5 points based on CRDQ score and −6 (only calculated for change in activities) based on 6MWD. Using the distribution-based methods, the estimates of MID ranged from −3 to −5 points for the different components.

**Conclusions:**

We concluded that the estimate of MID of the PFSDQ-M after pulmonary rehabilitation corresponds to a change of 5 points (range - 3 to −6) in each component in patients with severe COPD.

## Background

Patients suffering from chronic obstructive pulmonary disease (COPD) have low spontaneous levels of daily physical activity and impaired exercise performance [1]. They typically experience symptoms of dyspnea and fatigue when performing activities of daily life (ADL). Consequently, improving the patient’s functional status and symptoms during ADL is an important goal for treatment [1,2]. The modified Pulmonary Functional Status and Dyspnea Questionnaire (PFSDQ) is an instrument designed to quantify the experienced change in performing ADL compared with the period before disease onset and symptoms of dyspnea and fatigue related to ADL [3]. The components of the PFSDQ-M are assessed evaluating ten common activities, e.g. putting on a shirt, walk on inclines and climbing three stairs. In an era where patient-reported outcomes become increasingly important [4], a questionnaire that specifically evaluates the impact of the respiratory disease on activities of daily life appears to be an interesting tool. The magnitude of change after an intervention is difficult to interpret in the absence of the orientation on what constitutes an important difference for this patient reported outcome.

The minimal important difference (MID) of a specific instrument can be defined as “the smallest difference in score in the domain of interest which patients perceive as beneficial and which would mandate, in the absence of troublesome side effects and excessive cost, a change in the patient’s management” [5]. Determination of the MID is important for several reasons, as it facilitates judging the magnitude of the benefit when comparing two treatments, calculating sample sizes, making inferences about the percentages of patients improved by a therapeutic intervention (e.g. the number needed to treat), and making cost effectiveness comparisons [6]. Although no golden standard exists in the approach to calculate an MID, two global strategies have been proposed [7]. The anchor-based approach uses the relationship between the instrument under investigation and independent measures that target the same concept. In our situation, six-minute walking distance (6MWD) and Chronic Respiratory Disease Questionnaire score where used, as these anchors both evaluate aspects of activities of daily life. The distribution-based method interprets the magnitude of effect of the target instrument in relation to measures of variability.

The establishment of the MID for the PFSDQ-M would add to the interpretation of the improvement of dyspnea and fatigue symptoms and change in ADL after a pulmonary rehabilitation (PR) program [8,9]. The aim of this study was to establish the MID of the PFSDQ-M in patients with COPD using an anchor and distribution-based approach.

## Methods

### Subjects

Four hundred and sixteen patients with a clinical diagnosis of COPD participated in the outpatient PR program of the University Hospital Gasthuisberg in Leuven-Belgium in the period from March 2000 to July 2010. Patients were referred to the rehabilitation program with a clinical indication for pulmonary rehabilitation (poor exercise tolerance and disproportionate symptoms despite optimal pharmacotherapy). Baseline assessment was performed when patients were stable for at least four weeks. Exceptionally, some individual patients have been included earlier after the exacerbation based on advice of the pulmonologist. One-hundred fifteen patients did not complete the interviewed version of the PFSDQ-M before and after the PR program and were excluded, leaving a final sample 301 patients.

In addition, test-retest reliability was evaluated in 20 patients with COPD (FEV_1_ 52 ± 11%pred; 6MWD 510 ± 85 m) using a one-week interval between assessments. Baseline characteristics of both study samples are provided in Table 1.

**Table 1:** Baseline characteristics of the enrolled patients

**Variables**	**Mean ± SD**	**Test-retest analyses**
	**n = 301**	**n = 20**
**Gender (male/female)**	241/60	15/5
**Age (yrs)**	65 ± 7	68 ± 6
**BMI (kg/m**^**2**^**)**	25 ± 5	27 ± 6
**FEV**_**1**_**(% pred)**	42 ± 15	52 ± 11
**FEV**_**1**_**/FVC (%)**	39 ± 10	42 ± 9
**GOLD stage (I/II/III/IV)**	2/20/56/22	0/35/60/5
**FRC (% pred)**	156 ± 36	164 ± 37
**TLC (% pred)**	113 ± 18	124 ± 25
**TL,**_**CO**_**(% pred)**	47 ± 16	27 ± 6
**PImax (% pred)**	77 ± 26	75 ± 12
**VO**_**2**_**max (ml/kg/min)**	14 ± 6	14 ± 4
**W max (% pred)**	64 ± 25	78 ± 20
**6MWD (m)**	396 ± 124	510 ± 85
**PFSDQ-M activity (points)**	47 ± 17	29 ± 18
**PFSDQ-M dyspnea (points)**	48 ± 17	28 ± 20
**PFSDQ-M fatigue (points)**	42 ± 19	24 ± 20

*SD* standard deviation, *BMI* body mass index, *FEV*_*1*_ forced expiratory volume in one second, *FVC* forced vital capacity, *GOLD* Global Initiative on Obstructive Lung Disease, *FRC* functional residual capacity, *TLC* total lung capacity, *TL*_*CO*_ transfer factor of carbon monoxide, *PImax* maximum inspiratory pressure, *6MWD*, six minute walking distance, *PFSDQ-M* pulmonary functional status and dyspnea questionnaire modified version.

The study protocol was approved by the ethics committee of University Hospital Gasthuisberg, Leuven (project number B322201110245). Written informed consent was obtained from all patients.

### Study design

Data were retrieved from the PR database of the hospital where data at entry and follow up of PR were systematically recorded. The patients followed a 3-month multidisciplinary program including three exercise training sessions per week, according to international guidelines [10,11]. Before and after the training program, patients underwent an evaluation of lung function, respiratory and peripheral muscle force, maximal exercise capacity, 6-min walk distance (6MWD), health-related quality of life (CRDQ) and PFSDQ-M.

### Measurements pre and post pulmonary rehabilitation program

Static and dynamic lung volumes were measured according to the European Respiratory Society guidelines [12,13]. Diffusing capacity of the lung was assessed by the single breath method [14]. Maximal inspiratory pressure was measured from residual volume (MicroRPM; CareFusion, Basingstoke, UK) and was compared with reference values [15]. Functional exercise capacity was assessed using the 6MWD test. The best of two standardized tests was reported as percentage of the predicted value [16]. Maximal exercise capacity was evaluated using an incremental cycle ergometer (Ergometrics 900; Ergoline, Bitz, Germany). After 2 min of resting breathing, patients started a 3-min unloaded warm-up period. Subsequently work rate was increased by 10 W.min^-1^ until the symptom limited peak work rate was reached [17]. Oxygen uptake (VO_2_), carbon dioxide production (VCO_2_) and ventilation were measured breath-by-breath and averaged over 30 s (Vmax series; SensorMedics, Anaheim, CA, USA). The CRDQ was used to assess health-related quality of life [18]. This 20-item questionnaire scores quality of life in four domains (dyspnea, mastery, emotional functioning and fatigue) and has been validated in the Dutch language [19]. The total score can range from 20 to 140 with higher scores indicating better quality of life.

### Assessment of pulmonary functional status and dyspnea questionnaire – modified version

The PFSDQ-M consists of three components: dyspnea, fatigue and change in activities experienced by patients compared to the period before disease onset in 10 common activities [3]. The five general survey questions in the dyspnea and fatigue component components are considered informative and qualitative and these answers are not further analyzed. For each component, each activity is scored on an 11 point scale ranging from 0 to 10. Ratings on the dyspnea and fatigue component range from 0 “No shortness of breath/fatigue” to 10 “Very severe shortness of breath/fatigue” performing the specific activities. The change component is also rated on an 11 point scale from 0 “As active as I’ve ever been” to 10, “Have omitted entirely (the activity)”. Dyspnea component and fatigue component scores from 1–3 are labeled as mild, scores from 4–6 moderate and scores from 7–9 severe symptoms. Change component scores from 1–3 are labeled as a minor change, 4–6 a moderate change and 7–9 an extreme change in functional performance. For each of the three components a score ranging from 0 to 100 is calculated with lower scores indicating a better functional status. We also computed a total score (sum of scores in the 3 components) to divide patients in tertiles based on baseline functional status. The questionnaires were administered by the occupational therapist of the multidisciplinary pulmonary rehabilitation program as part of his baseline and follow-up assessment. It takes 10 to 15 minutes to complete the questionnaire.

### Pulmonary rehabilitation program

Patients attended the rehabilitation program three times per week. The multidisciplinary team consisted of a pulmonologist, an occupational therapist, a dietician, a psychologist, a social worker, a respiratory nurse and a team of experienced physiotherapists. An intake with all these professionals was scheduled at the start of the program and follow-up visits were scheduled as deemed necessary. Furthermore, all members of the team organized a group education session. Training sessions consisted of high-intensity aerobic training (treadmill walking, stationary cycle training, stair climbing and arm ergometry) and resistance training (upper and lower limbs). The session duration varied from 45 to 90 minutes. Aerobic training consisted of endurance training or interval training, when endurance training at an adequate training intensity was not feasible. Training intensity was increased gradually throughout the program. A target Borg score of four to six on perceived exertion or dyspnea was used to ensure optimal training intensity. Oxygen therapy was allowed during training to keep oxygen saturation above 90%.

The short- and long-term effects of our multidisciplinary rehabilitation program in terms of exercise tolerance and health-related quality of life have been reported previously [20].

### Anchor and distribution based methods

Different methods were used to determine the MID of the PFSDQ-M: one anchor-based method with two clinical anchors related to functional status (CRDQ and the 6MWD) and three distribution-based methods (Cohen’s effect size, empirical rule effect size and standard error of measurement (SEM) method).

#### Anchor based method

Using linear regression analyses, the known MID of the anchor was used to determine the corresponding minimal important change of the different PFSDQ-M components [21]. We used anchors that have been previously validated in patients with COPD: the CRDQ [22] and the 6MWD [22-25].

The CRDQ is a widely used instrument in respiratory rehabilitation used to assess health-related quality of life [26]. The twenty-item questionnaire scores quality of life in 4 domains which are dyspnea, fatigue, emotional functioning and mastery [18]. A change of 0.5 per item within each domain has been suggested as being the MID of the CRDQ [6,27,28]. Therefore if all questions within a domain are answered the clinically important difference for each domain is as follows: dyspnea 2.5, fatigue 2.0, emotional function 3.5 and mastery 2.0 points. A change of 10 points in the total score for the CRDQ is considered the MID. The interviewed Dutch version was used in this study [19]. The dyspnea and fatigue domains and the total score of the CRDQ were used as anchors.

The 6MWD is a standardized measure of functional exercise capacity in patients with COPD [29]. The test was performed in a 50 m long corridor of the hospital. Standardized encouragement was provided. The best result of two tests on separate days was used for analysis [16]. Changes in 6MWD are used to evaluate the efficacy of therapeutic interventions such as pulmonary rehabilitation [23]. The minimal important difference of the 6MWD test in patients with COPD has already been described in patients with different disease severity and using varying methodology [22-25]. In the present study, we opted to use the MID of 35 m, as proposed by Puhan et al., in our analysis [25]. This study investigated patients with comparable disease severity and used valid techniques to establish the MID, similar to our approach.

For the analyses we assessed the correlation between changes in the anchors and changes in the PFSDQ-M component scores. In addition, we used a linear regression analysis with PFSDQ-M component scores as the dependent and the anchors as independent variables if correlation coefficients were ≥ 0.3[22]. Using the regression equation and the MID of the anchors (0.5 points for each domain and 10 points for total score in the CRDQ and 35 m for the 6MWD) we estimated the MID of PFSDQ-M component scores.

#### Distribution based methods

We used the standard deviation (SD) of change in PFSDQ-M score after the rehabilitation program to calculate Cohen’s effect size and empirical rule effect size. According to Cohen, 0.5 × SD units represent a moderate effect size and investigators usually consider this estimate to correspond to an important effect [30]. The empirical rule effect size approach uses the empirical rule that in a normal distribution 99% of all observations lie within 3 SD below and above the mean. A change of 0.5 SD units corresponds to an 8% change within the normal distribution. According to the empirical rule effect size approach, 8% of the observed range (from the 0.5th to the 99.5th percentile) corresponds to an important effect. The SEM method multiplies SD of the baseline score with √ (1‒r), where r is the test-retest reliability coefficient (intra-class correlation coefficient (ICC)) of the PFSDQ-M [31].

Statistical analyses were performed with SAS 9. 2. V.2 statistical package.

## Results

Baseline characteristics of 301 patients used to assess the MID and 20 patients used to investigate test-retest reliability are reported in Table 1.

### Anchor-based approach

**Figure 1:**
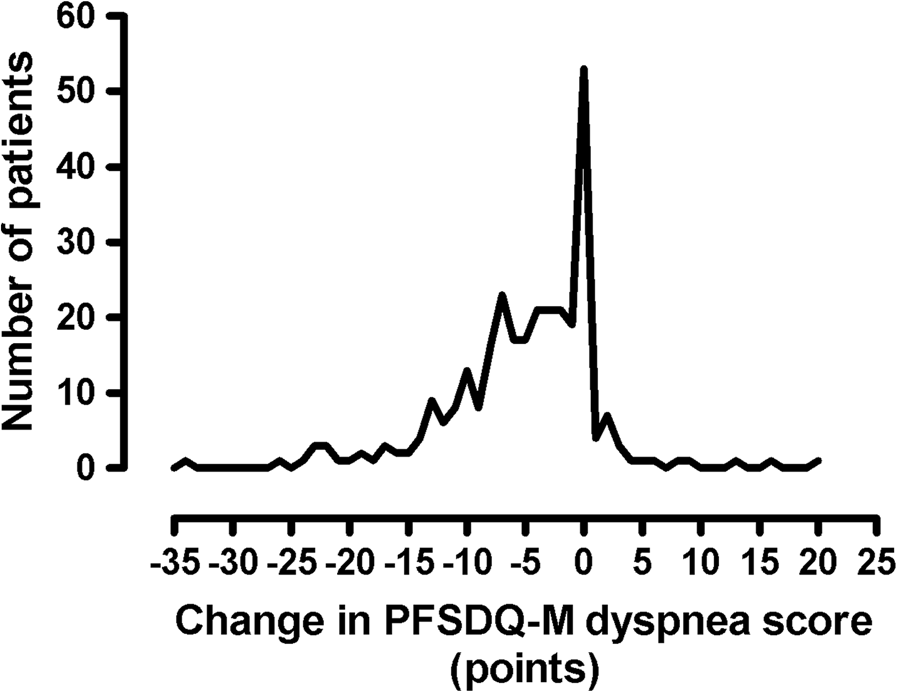
Frequency distribution of the change in PFSDQ-M dyspnea score after a 3-month pulmonary rehabilitation program.

**Figure 2:**
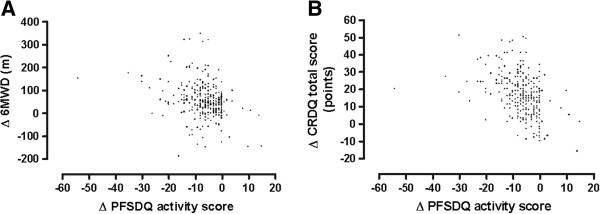
Relationship between changes in activity domain score of PFSDQ-M and 6MWD (2A; r = −0.30, p < 0.001) and CRDQ total scores (2B; r = −0.42, p < 0.001) after the rehabilitation program.

Table 2 shows baseline values and change after rehabilitation for PFSDQ-M and CRDQ scores and 6MWD. Changes in PFSDQ-M scores were independent of age, gender and baseline pulmonary function. The correlations between changes in PFSDQ-M component scores and changes in the anchors are also included in Table 2. Figure 1 provides the distribution of the change in PFSDQ-M dyspnea score after rehabilitation. Similar results were obtained for the other components. The change scores for the CRDQ and 6MWD both exceeded their established MID (0.5 point per item, and 10 points total score, and 35 meters, respectively). Correlations ≥ 0.3 were found between the change in PFSDQ-M components with change in CRDQ dyspnea and total score. Only the change in activity component of PFSDQ-M showed a correlation ≥ 0.3 with change in 6MWD (Table 2). The relationship between change in activity component scores and change in the anchors (6MWD and CRDQ total score) is illustrated in Figure 2. Correlations between change in PFSDQ-M components and change in the CRDQ fatigue domain were < 0.3. Table 3 shows the MID (95% CI) estimates based on the anchor-based method. The estimation of the MID was consistent across the seven regression models (correlation ≥ 0.3) [22] and ranged from −3.2 to −5.9 points. For the 6MWD Table 3 also provides the regression equation between PFSDQ-M and the 6MWD as there are several proposed thresholds for the MID of the 6MWD test. Using the regression equation ∆PFSDQ-M = −(0.03 * ∆6MWD) - 5.06 the MID of PFSDQ-M would be - 6,1 points using 35 m and −6.7 points using 54 m as MID of the 6MWD test. When applying the Cohen’s effect size method to our patient group, our estimate of MID of the 6MWD test would be 44.5, which corresponds with an MID of −6.3 points for the PFSDQ-M.

**Table 2:** Baseline values, changes and correlations of changes in PFSDQ-M and CRDQ scores and 6MWD from baseline to 3-months rehabilitation program

	**N = 301**		**PFSDQ-M activity component**	**PFSDQ-M dyspnea component**	**PFSDQ-M fatigue component**
	**Baseline values**	Δ**Changes from baseline to 3-month PR**	**- 6 ± 7**	**- 5 ± 7**	**- 5 ± 7**
**CRDQ dyspnea**	15 ± 4	6 ± 5	- 0.32*‡	- 0.30*‡	- 0.34*‡
**CRDQ fatigue**	15 ± 4	3 ± 3	- 0.27‡	- 0.23 ‡	- 0.21‡
**CRDQ total**	78 ± 16	16 ± 12	- 0.42*‡	- 0.41*‡	- 0.41*‡
**6MWD (m)**	396 ± 124	47 ± 89	- 0.30*‡	- 0.26‡	- 0.27‡

Data for changes are expressed in mean ± SD. *SD* standard deviation, *PR* pulmonary rehabilitation, *PFSDQ-M* pulmonary functional status and dyspnea questionnaire modified version, *CRDQ* chronic respiratory disease questionnaire, *6MWD* six minute walking distance.

Pearson correlation coefficient, ‡p < 0.0001. *Values indicate sufficient correlation for using the anchor based method (linear regression analyses).

**Table 3:** Anchor - based method to determine the minimal important difference of the PFSDQ-M

**Anchor**	**MID of PFSDQ-M (95% CI)**	**Score PFSDQ-M component**
CRDQ dyspnea score (MID =0.5 per-item × 5 items)	−5.0 (−6 to −3)	Activity
	−4.0 (−5 to −2)	Dyspnea
	−3.2 (−4 to −1)	Fatigue
CRDQ total score (MID = 10)	−4.9 (−7 to −2)	Activity
	−4.0 (−6 to −1)	Dyspnea
	−3.3 (−5 to −1)	Fatigue
6MWD (MID = 35 m)	−6,1 (−7 to −5)	Activity

*MID* minimal important difference, *PFSDQ-M* pulmonary functional status and dyspnea questionnaire modified version, *CRDQ* chronic respiratory disease questionnaire, *6MWD* six minute walking distance.

**Figure 3:**
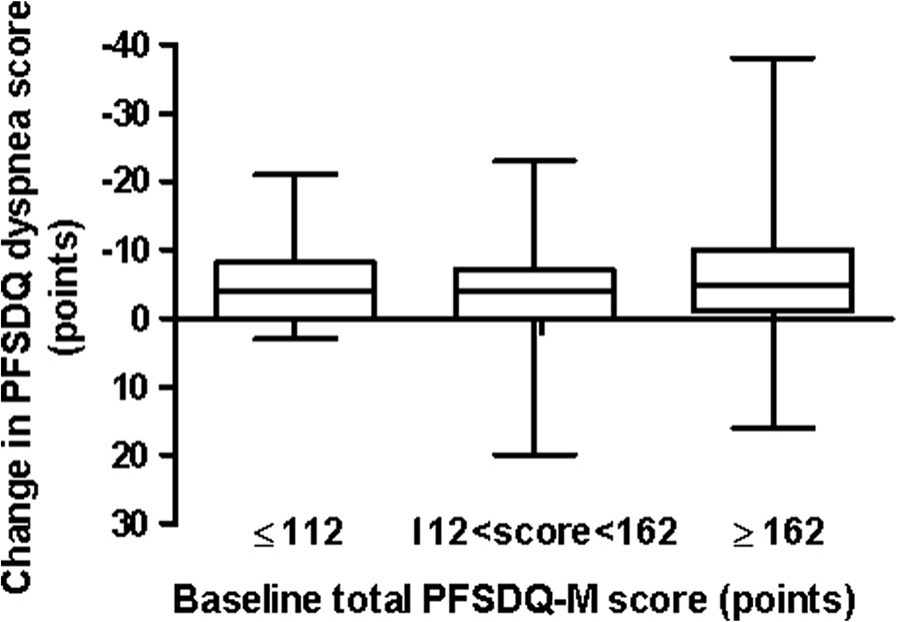
**Box plots of change in the dyspnea component of PFSDQ-M after 3 months of pulmonary rehabilitation related to baseline functional status.** Patients are divided in 3 tertiles based on baseline total PFSDQ-M score (lower tertile: ≤ 112; 112 < middle tertile < 162; higher tertile: ≥ 162 points; the lower tertile represents those patients with the best preserved functional status). No significant differences were observed between tertiles. Similar results were obtained for change in activities and fatigue components of PFSDQ-M.

To investigate whether changes in PFSDQ-M were influenced by the baseline functional status, we divided patients in three tertiles based on baseline PFSDQ-M total score (lower tertile: score ≤ 112; middle tertile: 112 < score < 162; higher tertile: ≥ 162) with the lower tertile representing those patients with the best preserved functional status. Changes in PFSDQ-M per tertile are shown in Figure 3 for the dyspnea component. Similar graphs are obtained for the change in activities and fatigue components. No significant differences in change of PFSDQ-M components were found between tertiles.

### Distribution-based approach

The ICC between test and retest was 0.79 for the change in activity component and 0.77 for dyspnea and fatigue components of PFSDQ-M. Table 4 shows the MID estimates using the distribution-based methods Cohen’s effect size, empirical rule effect size and SEM. The MID of PFSDQ-M using these techniques ranged from – 3.1 to – 4.7 points.

**Table 4:** Distribution - based method to determine the minimal important difference of the PFSDQ-M

**Distribution based method**	**MID of PFSDQ-M**
**Cohen’s effect size**	
Activity	−3.6
Dyspnea	−3.7
Fatigue	−3.5
**Empirical rule effect size**	
Activity	−4.0
Dyspnea	−4.3
Fatigue	−4.7
**Standard error of measurement**	
Activity	−3.2
Dyspnea	−3.3
Fatigue	−3.1

*MID* minimal important difference, *PFSDQ-M* pulmonary functional status and dyspnea questionnaire modified version.

## Discussion

This is the first study to determine the minimal important difference of the modified version of the PFSDQ in patients with severe COPD using different approaches based on state-of-the-art analytic techniques. Our estimates for the minimal important difference of the different PFSDQ-M components ranged from −3 to −6 points with anchor-based methods (using CRDQ dyspnea and total score and 6MWD as anchors) and from −3 to −5 points using different distribution-based methods (Cohen’s effect size, empirical rule effect size and the standard error of measurement method).

Anchor and distribution based method are conceptually very different [32]. Regardless of the apparent differences between methods, there is evidence that diverse methods yield apparently similar findings [33,34].

Despite the low correlation found between changes in the target instrument and changes of the anchors, the consistency of the result using different techniques to establish the MID and our large sample size [23,35] support the validity of our findings. As a consequence of the low correlation coefficient (< 0.3) [22] between the instruments we could not use the CRDQ fatigue domain as an anchor, neither the 6MWD for the PFSDQ-M dyspnea and fatigue component.

Estimates of the magnitude of clinically meaningful change in physical performance measures can contribute to the needs of clinical and research outcomes [35] of interventions. Functional status assessment is an important aspect in evaluating the patient’s ability to perform daily activities. The PFSDQ-M is a reliable and valid questionnaire [3,22]. In addition, it has been shown to be responsive to changes following pulmonary rehabilitation [22] and based on our data changes are independent of baseline functional status.

We suggest that when a group of patients decrease their PFSDQ-M score around 5 points this would be indicative of a clinically significant improvement of functional status. Estimations of the MID were similar in subgroups of patients with different baseline functional status. A change of −5 points corresponds approximately to a 12% change from the baseline scores and 5% of the range. This % of the range is in line with that observed in other questionnaires (e.g. SGRQ; MID of 4 points corresponds with 4% of the range) [36]. Other studies using other interventions may further fine tune the minimal important difference of the PFSDQ-M.

The PFSDQ-M is an instrument used in pulmonary rehabilitation that provides information about patient’s symptoms and their functional performance [3]. The meaningful change criteria of the PFSDQ-M appear achievable, because it is comparable with the magnitudes of improvement reported in research studies [37,38]. In addition, previous studies showed significant improvement in functional status after 3-month rehabilitation program [39,40], with decrease in PFSDQ-M scores that are in line with our results.

The estimates of change should be considered as preliminary evidence and will require further confirmation using similar as well as additional techniques. Patient ratings that could reflect perceived changes in exercise capacity and functional performance have also been used to identify minimal clinically important differences in previous studies [24,41,42]. However this technique may be biased by poor recollection and particularly changes in expectations when patients have to judge on themselves over a large period of time.

### Limitations of the study

Although we introduced original and new findings, the study has limitations which need to be addressed. First, this is a retrospective study over a long time period (eleven years). Although over this time period some changes in staffing were inevitable, the composition of the multidisciplinary team and the approach of the exercise training and the assessments have been consistent. The occupational therapist who administered the PFSDQ-M questionnaires was the same person throughout the study period. Second, the majority of included patients suffered from severe COPD, which prevents from generalizing our findings to all GOLD stages. Third, the Dutch version of the PFSDQ-M has not been validated. Although it is generally a translation of the original English version, the items brush hair and walk on bumpy terrain were replaced by putting on socks and shopping to optimize content validity. This adaptation was made with permission of the developers of the PFSDQ-M questionnaire.

### Clinical relevance

This study is the first to determine the MID of the modified version of the PFSDQ and provides a framework to judge impact of rehabilitation interventions on this patient reported outcome. Furthermore the established MID is a potential tool for clinical rehabilitation programs to assess efficacy of the program. It is however important to mention that the MID is not designed to evaluate training response in individual patients, due to the test-retest variability of the PFSDQ-M.

## Conclusions

The minimal important difference of the PFSDQ-M corresponds to a change of −5 points (range - 3 to −6 points) in patients with severe COPD on a scale ranging from 0 to 100 in each component. This estimate was confirmed by both anchor and distribution - based methods and seems relatively stable across baseline functional status. The PFSDQ-M is capable of capturing change in functional status over time. Further studies are necessary to evaluate whether the MID of PFSDQ-M remains stable in earlier stages of COPD.

## Abbreviations

ADL: Activities of daily life; COPD: Chronic obstructive pulmonary disease; CRDQ: Chronic respiratory disease questionnaire; FEV1: Forced expiratory volume in one second; ICC: Intra-class correlation coefficient; MID: Minimal important difference; SD: Standard deviation; SEM: Standard error of measurement; PFSDQ-M: Pulmonary functional status and dyspnea questionnaire – modified version; PR: Pulmonary rehabilitation; 6MWD: Six-minute walking distance.

## Competing interests

The authors report that no potential conflicts of interest exist with any companies/organizations whose products or services may be discussed in this article.

## Authors’ contributions

EMGR contributed to conceiving and designing the study, collecting the data, interpreting the data, writing the manuscript, and approving the final version of the manuscript. CB contributed to training the patients, conceiving and designing the study, collecting, analyzing and interpreting the data, providing critical revisions that are important for the intellectual content, and approving the final version of the manuscript. PB contributed to collecting the data, providing critical revisions that are important for the intellectual content, and approving the final version of the manuscript. DL contributed to training the patients, collecting the data, interpreting the data, providing critical revisions that are important for the intellectual content, and approving the final version of the manuscript. HVR contributed to training the patients, collecting the data, interpreting the data, providing critical revisions that are important for the intellectual content, and approving the final version of the manuscript. VAPD contributed to providing critical revisions that are important for the intellectual content, and approving the final version of the manuscript. DC contributed to providing critical revisions that are important for the intellectual content, and approving the final version of the manuscript. WJ contributed to interpreting the data, providing critical revisions that were important for the intellectual content, and approving the final version of the manuscript. MD contributed to interpreting the data, providing critical revisions that were important for the intellectual content, and approving the final version of the manuscript. RG contributed to conceiving and designing the study, interpreting the data, providing critical revisions that are important for the intellectual content, and approving the final version of the manuscript. TT was senior investigator, contributed to conceiving and designing the study, collecting the data, interpreting the data, writing the manuscript, and approving the final version of the manuscript. He also performed the statistical analysis. All authors read and approved the final manuscript.
